# Lupus Nephritis With Collapsing Glomerulopathy: A Rare Association

**DOI:** 10.7759/cureus.45215

**Published:** 2023-09-14

**Authors:** Shubham Dubey, Pranjal Kashiv, Kapil N Sejpal, Prasad Gurjar, Sushrut Gupta, Vrushali Mahajan, Amit Pasari, Manish Balwani

**Affiliations:** 1 Department of Nephrology, Jawaharlal Nehru Medical College, Datta Meghe Institute of Higher Education and Research, Wardha, IND; 2 Department of Pathology, Alexis Multispecialty Hospital, Nagpur, IND

**Keywords:** end stage renal disease (esrd), masson’s trichrome stain, rapidly progressive renal failure, crescentic gn, systemic lupus erythematosus, collapsing glomerulopathy, posterior reversible encephalopathy syndrome (pres), podocytopathy, lupus nephritis, collapsing glomerulonephropathy

## Abstract

Lupus nephritis (LN) is one of the most severe organ manifestations of systemic lupus erythematosus (SLE). Crescentic lupus nephritis rarely presents as rapidly progressive renal failure (RPRF) and needs prompt initiation of treatment. Collapsing glomerulopathy (CG) itself is associated with poor renal survival. Collapsing glomerulopathy's association with lupus nephritis is rarely reported in the literature. It may indicate a severe form of lupus podocytopathy.

## Introduction

Lupus nephritis is a common and serious complication in patients with systemic lupus erythematosus (SLE), developing in almost 50% of cases of SLE [1,2]. In the majority of patients, LN develops within 5 years of diagnosis of SLE [3].

Severe forms of LN (like crescentic lupus and lupus with thrombotic microangiopathy) can present with rapidly progressive renal failure (RPRF). The International Society of Nephrology (ISN)/Renal Pathology Society (RPS) system classifies LN into six different types based on glomerular histology using light and immunofluorescence microscopy: minimal mesangial LN, mesangial proliferative LN, focal LN, diffuse LN, membranous LN, and advanced sclerosing LN [4]. There are many histologic appearances of LN on light microscopy. However, LN with collapsing glomerulopathy (CG) is rarely reported in the literature. Hence we report a case of CG with LN in a patient with RPRF.

## Case presentation

A 32-year-old female presented with abdominal distension and facial puffiness since one month, shortness of breath since 10 days, and decreased urine output since 7 days. There was no prior history of hypertension, diabetes mellitus, cardiac ailment, or any other comorbidities. On examination, the patient had mild pallor and facial puffiness. Heart rate was 80/min, BP 160/100 mm Hg, respiratory rate 17/min, mild ascites, and bilateral end-inspiratory basal crepitations. Investigations showed severe renal failure and proteinuria. Further workup showed antinuclear antibody (ANA) positivity and low serum C3 level (Table 1).

**Table 1:** Laboratory investigations ANA: antinuclear antibodies

Investigations	Results	Reference Range
Hemoglobin	9.7 gm/dl	12-16 gm/dl
Total leucocyte count	4700/cumm	4000-11000/cumm
Platelets	242000/cumm	150000-450000/cumm
Blood urea	127 mg/dl	7-17 mg/dl
Serum creatinine	5.9 mg/dl	0.52-1.04 mg/dl
Serum sodium	132 mmol/l	137-145 mmol/l
Serum potassium	5.3 mmol/l	3.5-5.1 mmol/l
Urine albumin	3+	Nil
Urine RBCs	Nil	Nil
Urine pus cells	Nil	Nil
ANA	Positive	<0.9 (Negative), 0.9-1.1 (Borderline Positive), >1.1 (Positive)
Serum C3	46 mg/dl	80-165 mg/dl

**Figure 1:**
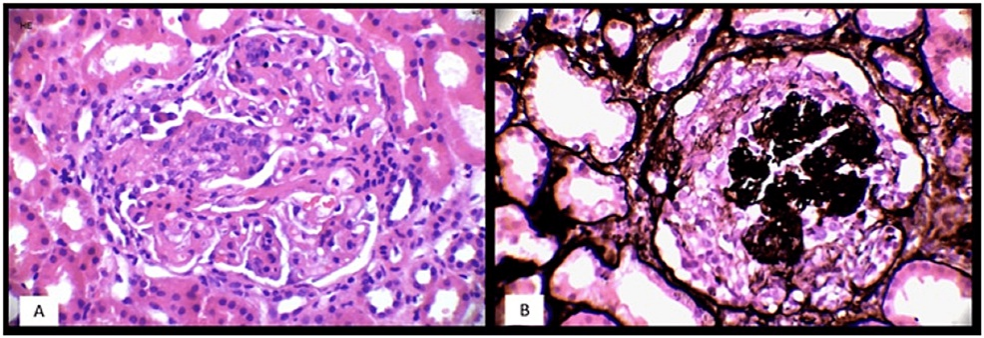
A - Microphotograph showing proliferative glomerulonephritis (Hematoxylin & Eosin stain 40X), B - Microphotograph showing collapsing pattern of glomerular injury (Jones Methenamine Silver stain 40X)

**Figure 2:**
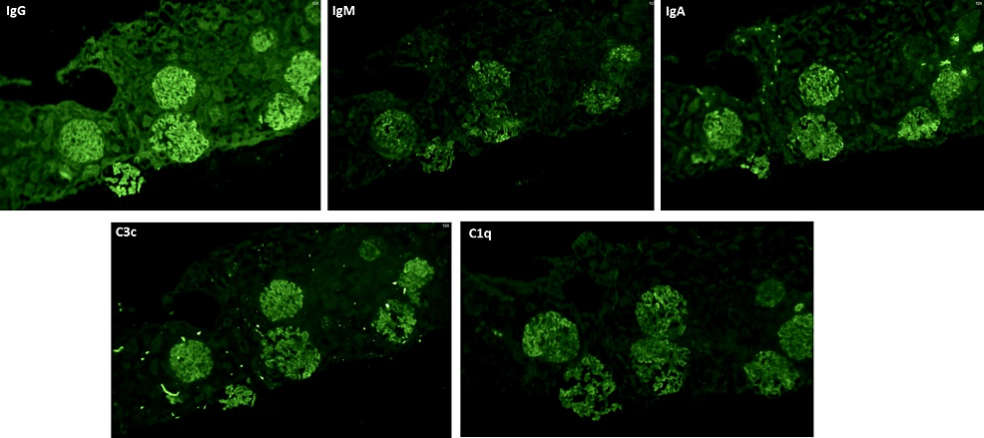
Immunofluorescence findings - Full house immune deposits in glomeruli with deposits of three immunoglobulins (IgG, IgM, IgA) and two complement components (C3c, C1q)

**Figure 3:**
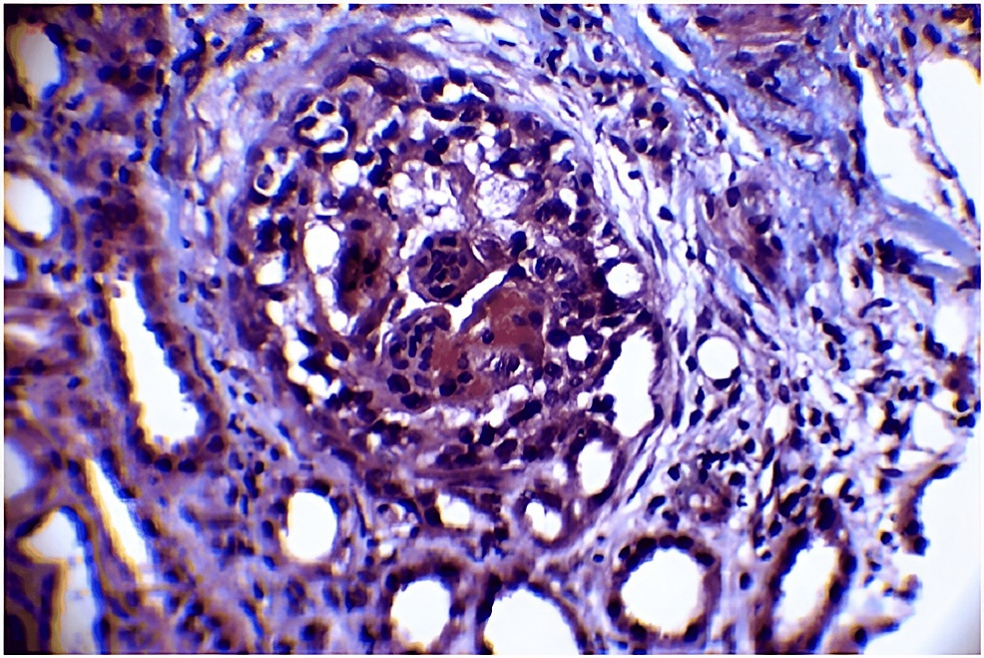
Masson's trichrome stain 40 X microphotograph showing collapsed hypercellular glomerular tuft with hyperplastic visceral epithelial cells

In view of severe uremia, she underwent two sessions of hemodialysis followed by ultrasonography-guided native kidney biopsy. The biopsy was suggestive of crescentic (18/25) class IV lupus nephritis with a collapsing pattern of glomerular injury (Figures 1-3). Activity score was 15/24 and chronicity was 1/12. Electron microscopy could not be done due to logistic reasons.

**Figure 4:**
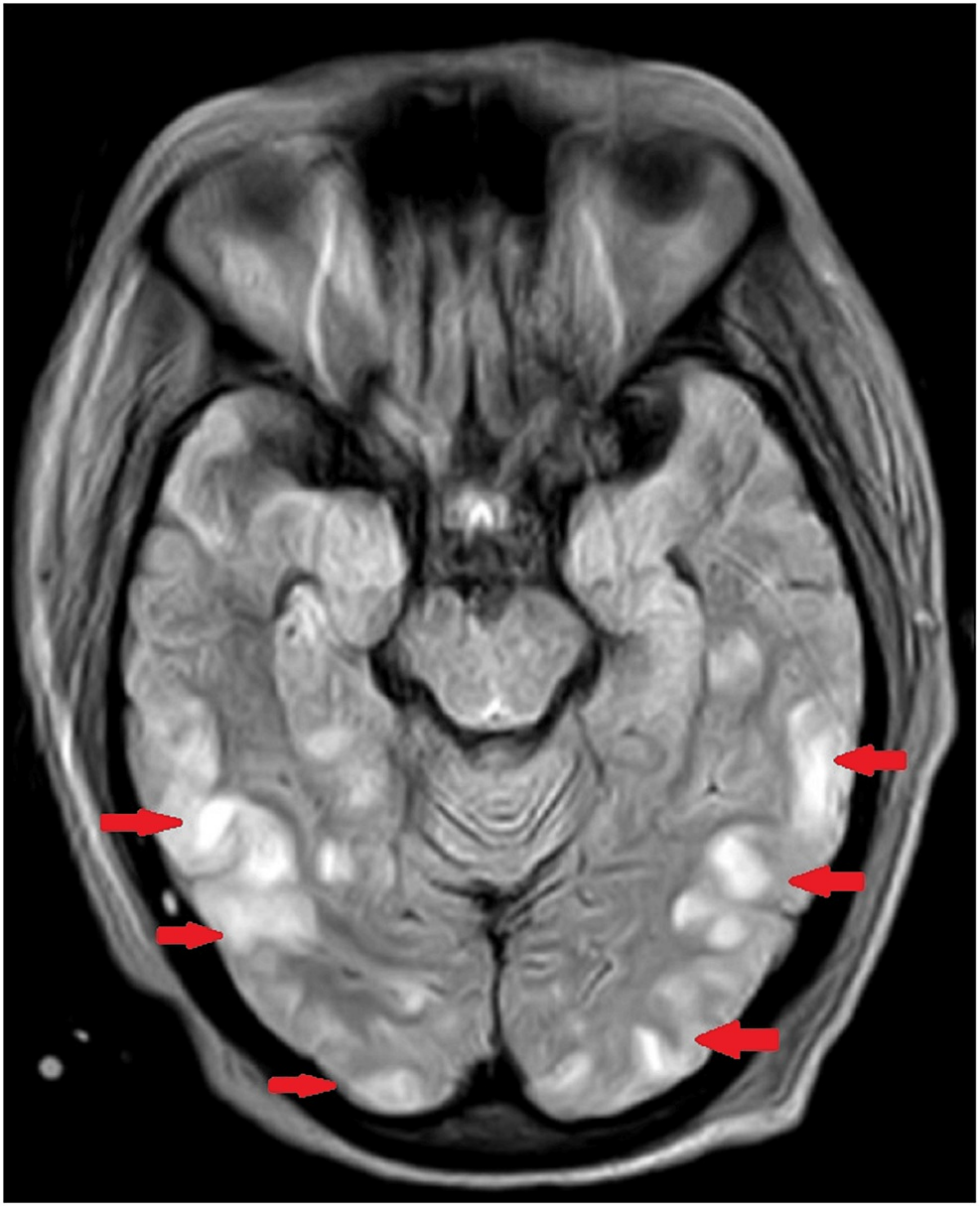
Bilateral parietal, temporal, and occipital white matter signal hyperintensities on MRI Brain T2/FLAIR sequence (red arrows) FLAIR: fluid-attenuated inversion recovery

HIV, Hepatitis B, and Hepatitis C were negative. A COVID-19 test in view of CG was done, which was negative. The patient developed one episode of generalized tonic-clonic seizure associated with accelerated hypertension. MRI brain was done and was suggestive of multiple altered signal intensity areas in the bilateral frontal, parietal, temporal, and occipital regions, involving cortical and subcortical areas and medial thalami, bilateral periaqueductal grey matter, and bilateral cerebellar hemisphere. It was hyperintense on T2/FLAIR (fluid-attenuated inversion recovery) sequences showing facilitated diffusion on Apparent Diffusion Coefficient (ADC), showing no blooming on Susceptibility-Weighted Imaging​​​​​​​ (SWI)suggestive of posterior reversible encephalopathy syndrome (PRES) (Figure 4). The patient was managed conservatively with strict BP control, after which there was no recurrence of seizures. 

The patient was pulsed with an injection of methylprednisolone, and an injection of cyclophosphamide 750 mg was given as an induction agent. There was no significant improvement in her urine output after 10 days of treatment, hence she was offered plasma exchange as a rescue therapy. She received two cycles of plasma exchange. Her renal function did not improve despite all these measures during the 25 days of hospital stay. She was discharged with advice for maintenance hemodialysis and close follow-up in nephrology OPD.

## Discussion

Lupus nephritis (LN) is a form of glomerulonephritis which is one of the most severe organ manifestations of SLE [5]. The Systemic Lupus International Collaborating Clinics (SLICC) criteria include biopsy-proven nephritis compatible with LN in the presence of either antinuclear antibody (ANA) or anti-double stranded (anti-ds) DNA antibody as sufficient evidence for the diagnosis of SLE. The incidence and prevalence of lupus and LN are influenced by age, gender, ethnicity, geographical region, diagnostic criteria used, and method of ascertainment, and across populations, clinically important kidney disease occurs in about 50% of patients with SLE [1,2]. The peak incidence of lupus is between the ages of 15 and 45 years, with a women to men ratio of 8-15:1. Amongst the patients with lupus, it affects both sexes equally, and is more severe in men and children, and is lesser in older adults. The incidence of LN is about 30% in White, 60% in Black and Hispanic, and 40% to 80% in Asian patients with SLE [1,6]. Activity scoring of SLE is done by the SLE Disease Activity Index (SLEDAI) [7,8] or the British Isles Lupus Assessment Group Index (BILAG 2004) [9].

Collapsing glomerulopathy is a pattern of glomerular injury, characterized by severe injury to podocytes associated with loss of differentiation markers, proliferation of the podocytes and/or parietal epithelial cells which fill the Bowman’s space, and segmental or global collapse of capillary tuft [10-13]. The various etiologies of CG include viral infections (most commonly HIV, hepatitis C, and parvovirus B19), genetic mutations, drugs, vascular occlusion, and other idiopathic causes. CG in conjunction with SLE has some clinical, demographic, immunohistologic, and morphologic features with HIV-Associated Nephropathy(HIVAN). These include association with African descent persons; a characteristic clinical presentation with heavy proteinuria and renal insufficiency often present; a frequent progression to end-stage renal disease (ESRD), without the presence of treatment-induced remission of proteinuria; tubulointerstitial scarring and tubular injury along with the characteristic glomerular lesions of CG; loss of podocyte differentiation markers in the glomeruli with CG lesions and proliferation of the glomerular epithelial cells [14].

The relationship between CG lesions and lupus podocytopathy remains unclear, much like with non-collapsing and collapsing forms of idiopathic focal segmental glomerulosclerosis (FSGS), the primary target of injury in all of these lesions is possibly the podocyte, and CG thus may indicate an extreme form of the lupus podocytopathy.

## Conclusions

Collapsing glomerulopathy with lupus nephritis is a rare presentation. The exact etiology remains unknown, however, it is assumed that it is a form of lupus podocytopathy. Little data is available regarding this entity and its progression to ESRD.
